# AI-Powered Socially Assistive Robots: Revolutionizing In-Home Therapeutic Exercise for Older Adults

**DOI:** 10.1093/geroni/igaf122.1544

**Published:** 2025-12-31

**Authors:** Dain LaRoche, Momotaz Begum, Sajay Arthanat, Jing Wang, Paul Gesel

**Affiliations:** University of New Hampshire, Durham, New Hampshire, United States; University of New Hampshire, Durham, New Hampshire, United States; University of New Hampshire, Durham, New Hampshire, United States; University of New Hampshire, Durham, New Hampshire, United States; PickNik Robotics, Boulder, Colorado, United States

## Abstract

Physical activity for older adults is an important countermeasure to reduce the risk for chronic disease, falls, osteoporosis, depression and dementia while serving to preserve mobility and independence in activities of daily living. Access to structured physical activity and rehabilitation services is hampered by a deficit of licensed physical and occupational therapists, transportation challenges, and proximity to care services, particularly for older adults with disability and those who live in rural settings. Artificially intelligent SAR have the potential to bridge the care access gap by providing home exercise programs that can be tailored and autonomously delivered to older adults. A learning from demonstration algorithm allows therapists to teach robots exercises without computer programming, and computer vision can be used to recognize, quantify, and assess the quality of the exercises performed by older adults in their home, allowing for the identification of movement deficiencies and tracking of progress over time. We will illustrate the process by which therapists can teach robots exercises using AI, how the exercises are encoded and delivered by the robot, a framework for tracking performance, and results of a survey of 110 licensed therapists who were asked to evaluate the potential for robot-led therapy in home settings. We will discuss the emerging potential of SAR as an in-home adjunct therapy to supplement clinical exercise programs as well as the barriers to implementation including the anatomical structure of current robots, ability to provide real-time, physical, corrective feedback, potential loss of human connection, user acceptance and cost.

